# The effect of 12 weeks of water-aerobics on health status and physical fitness: An ecological approach

**DOI:** 10.1371/journal.pone.0198319

**Published:** 2018-05-31

**Authors:** Henrique Pereira Neiva, Luís Brandão Faíl, Mikel Izquierdo, Mário C. Marques, Daniel A. Marinho

**Affiliations:** 1 Department of Sport Sciences, University of Beira Interior, Covilhã, Portugal; 2 Research Center in Sports Sciences, Health Sciences and Human Development, CIDESD, Vila Real, Portugal; 3 Department of Health Sciences, Public University of Navarre, CIBER of Frailty and Healthy Aging (CIBERFES; CB16/10/00315), Navarrabiomed, Pamplona, Navarre, Spain; Texas A&M University, UNITED STATES

## Abstract

The main purpose of the present study was to verify the effects of a 12-week water aerobics program in a real-life context on health indicators and physical fitness in adults and older adults. Fifteen volunteers (58.80 ± 14.32 years old) were part of an experimental group (Exercise), and eight volunteers (59.00 ± 12.26 years old) were part of the control group (Control). The Exercise performed 45 min of water aerobics twice a week for 12 weeks; no physical exercise was permitted for the Control during the same period. The evaluations were performed the week before (pre-training) and after the training program (post-training). The primary outcomes were the strength and cardiorespiratory fitness variables and the secondary outcomes included body anthropometry, lipid profile and blood pressure. Adjusted analysis for age and baseline values showed no differences between Exercise and Control in post-training moment. However, there was a moderated tendency for increased explosive strength of the upper limbs (η_p_^2^ = 0.17), reduced body fat (η_p_^2^ = 0.17), reduced systolic blood pressure (η_p_^2^ = 0.14) and triglycerides (η_p_^2^ = 0.19) in Exercise. Within groups changes showed that the training program caused an increase mainly in explosive strength in the upper limbs (0.26 m, 95% CI, 0.03, 0.49; ES = 0.63) In addition, there was a significant decrease in fat mass (-0.89%, 95% CI, -1.74, -0.03; ES = 0.61) and in the systolic blood pressure (-0.83 mmHg, -1.46, -0.19; ES = 0.71). Nonetheless, no significant changes were observed for the lipid profile. These results suggest that 12 weeks of water aerobics performed twice a week in a real-life context seem to benefit the explosive strength, body composition, and blood pressure of adults and older adults but is insufficient to alter cardiorespiratory fitness and lipid profiles.

## Introduction

Over the years, there has been a widespread recommendation for all people to participate in physical activity on a regular basis. However, certain limitations may restrict people’s ability to participate in exercise programs, specifically obesity, low levels of physical fitness, locomotion difficulties caused by aging, orthopedic or neurological disabilities, or pulmonary disease [1, 2]. Given these difficulties, health and sports professionals have recommended water-based exercises as an alternative to traditional dry-land exercise, leading to a significant increase in physical exercise performed in an aquatic context [1]. The properties of the aquatic environment, which reduce the effect of body weight on the joints and compression forces and reduce the risk of injury or fall, combined with the resistance of the water during all movements, make it beneficial for overall body exercise and recovery from injuries [2–5].

Currently, water aerobics is among the aquatic programs most widely recognized by health specialists, sport professionals, and practitioners [6, 7]. This recognition could be due not only to the organic changes caused by hydrostatic pressure, buoyancy and thermodynamics but also to the variety of movements that can be carried out using the properties of water to create resistance to movement with reduced neuromuscular activity required from the antigravity muscles [6, 8]. These aspects could be used to improve the physical conditions of people with certain difficulties as well as healthy young people and adults [9]. Studies have reported improvements in oxygen uptake [10, 11], muscle strength [12, 13] and body composition [13, 14] as a result of water aerobics participation. Nevertheless, previous discussions have noted that the exercise program should be specific enough or long enough to cause effective improvements [15]. Studies with a short duration and lower intensities, even with experienced practitioners, have found contrary results [6], and further research should be developed.

The most frequently studied chronic changes during aquatic exercises are those that affect the cardiovascular system, muscle strength and body composition. These adaptations are strongly related to individual health and fitness [6]. Exercise programs should focus on these parameters to induce favorable adaptations of total cholesterol, triglycerides, and other relevant variables related to coronary artery disease [16]. Few studies have analyzed the responses of glycemia, lipoproteins and lipids profiles to aquatic exercise, and differing results impede clear conclusions regarding this subject [17]. Moreover, and despite the general approval by the sports and health communities, the scientific evidence is not consistent regarding the value of water aerobics for improving and/or maintaining health and physical fitness in sedentary populations and those with special needs [17, 18].

Although it appears that water-based exercise may be suitable for adults and older individuals, the studies that have examined this relationship had methodological flaws, such as the possible physiological effect of water immersion, the range of intensities and durations, and differences in program designs, which raise doubts regarding their real effects. Nevertheless, most programs are designed by researchers and/or use few exercises, and studies of what occurs in a real-life venue still lacking. Most studies were implemented based on external validity, with extremely controlled environments that do not represent real-life situations. There is a real need for understanding the effects of the different programs that are usually implemented in sports academies. Thus, the primary aim of the current study was to verify the effects of a 12-week water aerobics program performed on a real-life context of sports academy on physical fitness and health indicators in adults and older adults. The primary outcomes for our study are the strength and cardiorespiratory fitness. Secondary outcomes included health-related variables, such as body anthropometry, lipid profile and blood pressure. We hypothesized that 12 weeks of training would increase explosive and endurance strength of lower and upper limbs and overall cardiorespiratory fitness. It was also hypothesized to have positive effects on health by reducing body fat, triglycerides, cholesterol and blood pressure.

## Materials and methods

### Participants

The subjects were recruited from the same residential zone and participants in water aerobics classes at the same sport academy. Participants of different classes, randomly chosen, were informed about the study protocol, risks, and benefits and once they agreed, they voluntarily signed the informed consent form. The procedures were performed according to the Declaration of Helsinki and were approved by the review board of Research Center in Sports Sciences, Health Sciences and Human Development of the University of Beira Interior, Portugal.

For inclusion in the experimental group (Exercise), the individuals had to i) be water aerobics practitioners for more than six months; ii) participate in at least two lessons per week regularly; iii) be aged 18 years or older. The control group (Control) included subjects older than 18 years who did not exercise regularly and living at the same residential zone of the sports academy. Subjects were excluded from the study if they presented a recent hospitalization, severe cognitive or motor impairments, an inability to exercise and any other medical contraindications for physical exercise. Prior to the starting the intervention, the sample size calculation for an unmatched case-control study was determined using OpenEpi (OpenEpi, Version 3, open source calculator—SSPropor) and a minimal of 10 subjects and 6 subjects should be allocated to Exercise and Control, respectively, to obtain a power of 0.8 at a two-sided level of 0.05.

An initial sample of 31 individuals were assessed for eligibility and 29 agreed to take part in the study. From these, twenty-three subjects were included upon meeting the criteria for selection. The Exercise included 15 participants of both sexes, two males and thirteen females (58.80 ± 14.32 years of age; 1.61 ± 0.07 m of height; 72.54 ± 15.53 kg of body mass; 6.93 ± 5.70 years of experience in water aerobics). All of them participated in water aerobics classes at the same sports academy. The Control included eight subjects, two males and six females (59.00 ± 12.26 years of age; 1.62 ± 0.08 m of height; 70.71 ± 8.66 kg of body mass). The Control did not participate in the water aerobics classes and maintained their basic daily activities without physical exercise (for details on flow diagram of study enrollment: http://dx.doi.org/10.17605/OSF.IO/6R5UW).

### Procedures

The present study consists of a non-randomized controlled trial that aimed to verify the changes in physical fitness (explosive strength and strength endurance of the lower and upper limbs, cardiorespiratory fitness) and health status (body anthropometrics, cholesterol levels, triglycerides, blood pressure) and after 12 weeks of water aerobics lessons. These variables were assessed during the week before the program implementation (week 0—pre-training) and the week after the end of the program (week 13—post-training). The variables assessed could be categorized into different groups, namely, i) anthropometry, with the measurement of height, body mass by bioimpedance, and circumferences (waist and hip); ii) physical condition, through the evaluation of the explosive strength of the upper limbs (3-kg medicine ball throwing) and lower limbs (countermovement jump), endurance strength of the upper limbs (maximum number of arm pushups) and the lower limbs (chair -stand test) and cardiorespiratory fitness evaluation (YMCA 3-min step-test); iii) lipid profile (triglycerides and cholesterol) and blood pressure.

The variables were evaluated on two different days separated by 72 hours. On the first day, the participants’ anthropometric characteristics, lipid profile and blood pressure were measured, followed by an assessment of explosive strength of the upper and lower limbs. On the second day of assessment, endurance strength and cardiorespiratory tests were performed. When the physical condition was assessed, the participant was allowed to rest for at least 30 min to fully recover from the previous evaluation. The subjects were instructed in advance to refrain from exercise, alcohol and caffeine consumption during the evaluation period. All variables were assessed by experienced researchers. They were not informed about the group to which the subjects belonged to. (for details on protocol: http://dx.doi.org/10.17504/protocols.io.ncudaww).

#### Physical condition

The explosive strength of the upper limbs was evaluated using a 3-kg medicine ball throw. This measurement was performed according to the protocol described in the literature [19]. Each participant was seated on the ground and instructed to launch the 3-kg medicinal ball (Vinex, model, VMB-003R, perimeter, 0.78 m) from the chest so that it traveled as far as possible before it touched the floor. For each of three repetitions, the distance from the starting position of the ball to the point at which it fell was measured. To evaluate the explosive strength of the lower limbs, the participants performed a countermovement jump (CMJ) on an OptoJump platform (Ergojump, Globus Italia, Codogné, Italy). From a standing position with the feet shoulder- width apart and the hands placed at the waist, the subjects performed a quick countermovement with the lower limbs before jumping. They were instructed to jump vertically as high as possible. Each participant performed three jumps with 1 min of recovery between attempts. The mean and maximal values for the ball throwing and CMJ tasks were considered for further analysis.

The Chair Stand Test [20] was used to assess the endurance strength of the lower limbs. The test began with the participants sitting in the chair, with back straight and feet spread apart at the shoulder-width and fully resting on the ground. The upper limbs were crossed at the level of the wrists and against the chest. At the starting signal, the participant should get up from the chair to the vertical position and then return to the initial seated position, repeating as many repetitions as possible for 30 seconds. The endurance strength of the upper limbs was obtained by assessing the maximum number of arm pushups until exhaustion. For the repetition to be considered valid, the participant would have to ensure that the arm was parallel to the ground, with the angle between the arm and forearm being approximately 90°. If there were two errors of execution or body posture, during the execution, the evaluation would cease. Because of physical limitations, five of the Exercise subjects and one of Control subjects did not perform the arm pushups.

The evaluation of cardiorespiratory fitness assessment was performed using a three minutes step test in accordance with the YMCA protocol [21]. Each participant performed the alternative rise of the lower limbs to a step with the height of 30 cm with a pace of 94 beats per minute by a beep sound. Heart rates were recorded immediately after the 3-minute test duration. These values were used to analysis to avoid any error associated with the indirect determination for the maximum oxygen uptake by using indirect equation associated with this test.

#### Anthropometrics

All anthropometric measures were evaluated according to international standards for anthropometric evaluation [22] and before any physical performance test was initiated. The participants were barefoot and dressed in as little clothing as possible for the evaluations. To measure body height, a precision stadiometer with a scale of 0.001 m was used (Seca 213, Hamburg, Germany). Body mass index was obtained by dividing the body mass value by the height squared (kg/m^2^). The waist circumference was measured using a tape measure placed around the waist along the horizontal plane, 1 cm above the top of the iliac crests and without compressing the skin. After the subject exhaled fully, the circumference was recorded to a precision of 0.1 cm; measurements were repeated until the error did not exceed this value. Using the same guidelines, the hip circumference was assessed with the tape placed at the widest area of the hip. Later, the waist/hip ratio was calculated [23]. Body composition was determined using a four-electrode bio-impedance analysis for each subject (Tanita, BC418 MA, Tokyo, Japan) that allowed the assessment of body mass, body fat, and fat-free mass.

#### Lipid profile and blood pressure

Capillary blood samples for cholesterol and triglyceride assessment were collected from fingertip (Accutrend Plus, La Roche, Germany) at rest and using lancets and strips specific to the equipment. The blood pressure was measured in the sitting position, after resting for 20 min and following the standards for the effect using an automated non-invasive blood pressure monitor (OMRON M4-1, Hoofddorp, Netherland) [21].

#### Water aerobics lessons

The water aerobics lessons held during the 12-weeks program were the ones usually performed at the institution. The subjects performed the lessons routines, twice a week, each lone lasting 50 min. The researchers did not interfere with the water lessons programming, evaluating only the usual classes carried out by the teacher. Each lesson started with a warm-up (e.g., jogging and lateral movements) of 8 ± 2 min (91 ± 3 bpm; 54 ± 3% of age-predicted maximal heart rate). Then, it was followed a main routine divided in 27 ± 2 min of aerobic exercitation, with all body stimulation (e.g., general exercises for upper and lower limbs simultaneously performed), recording heart rates between 101 and 126 bpm (112 ± 7 bpm; 66 ± 5% of age-predicted maximal heart rate), and 10 min of specific exercitation (e.g., specific upper or lower body exercitation), recoding heart rates between 98 and 118 bpm (109 ± 6 bpm; 64 ± 4% of age-predicted maximal heart rate). The lessons ended usually with approximately 5 min of active recovery, with heart rates between 90 and 99 bpm (94 ± 2 bpm; 55 ± 3% of age-predicted maximal heart rate). The swimming pool where the water aerobics took part was 1.50 m deep and the water temperature approximately 29°C.

#### Statistical analysis

Standard statistical procedures were selected to calculate means, standard deviations (SD) and 95% confidence limits. For the statistical analysis between groups, we made several analysis decisions relative to our study population that entailed adjusting our analysis for covariates that might influence the overall outcome. These included the age and each respective parameter baseline. Despite a lack of difference between groups at baseline, the age range was quite broad and should be accounted for. In addition, the baseline values of each parameter could influence final results, even more when the intervention groups were from different sample sizes. We did not control for gender, since almost all were females.

The normality and homogeneity of variance were confirmed by using Shapiro-Wilks and Levene test. Independent samples t-test was used to compare dependent variables at baseline between groups. Then, general linear models adjusted for the aforementioned parameters were used to compare changes form pre- to post-training moments between groups, by Bonferroni’s post hoc comparison tests. Decisions on within group changes were considered significant at the 0.05 level if accompanied by 95% CI that did not cross zero. Moreover, Student’s paired t-test was used for repeated measurements (pre vs. post) in each group to deepen the analysis. All these statistical procedures were performed using IBM SPSS Statistics for Windows^®^, version 22.0. (Armonk, NY, USA: IBM Corp.) and the level of statistical significance was set at p ≤ 0.05. In addition, the effect size was calculated to estimate variance between conditions (partial eta squared: η_p_^2^) and Cohen’s *d*_z_ (ES) for within-subjects’ comparisons using the Excel spreadsheet by Lakens [24]. ES values between 0.20 and 0.50 were considered small, between 0.50 and 0.80 were considered medium, and ≥ 0.80 were considered large [24]. For η_p_^2^, cut-off values were interpreted as 0.01 for small, 0.09 for moderate and 0.25 for large.

## Results

In the beginning of the training program, there were no significant differences between Control and Exercise in age (p = 0.97), anthropometric characteristics (height: p = 0.95; body mass: p = 0.76; body mass index: p = 0.73; fat-free mass: p = 0.38; waist circumference: p = 0.72; hip circumference: p = 0.72; waist/hip ratio: p = 0.87), lipid profile (triglycerides: p = 0.74; cholesterol: p = 0.22) and blood pressure (systolic: p = 0.57; diastolic: p = 0.57). In addition, no significant differences were found in physical condition between groups (explosive strength of upper limbs: p = 0.18; endurance strength: p = 0.17 and p = 0.14, for lower and upper limbs; YMCA heart rate: p = 0.22), excepting for the explosive strength of the lower limbs (mean values: p = 0.02).

After adjusting for age and baseline values, the variables assessed to evaluate physical condition showed only moderated effect sizes between Exercise and Control in post-training for ball throws (mean-values: *F* = 3.27, p = 0.09, η_p_^2^ = 0.15; maximal-values: *F* = 3.78, p = 0.07, η_p_^2^ = 0.17). Neither of the main differences between groups in the other physical condition variables were significant or relevant, countermovement jump: *F* = 0.69, p = 0.42, η_p_^2^ = 0.04 for mean-values, *F* = 0.72, p = 0.41, η_p_^2^ = 0.04 for maximal values; endurance strength of the lower limbs: *F* = 0.08, p = 0.78, η_p_^2^ = 0.004; endurance strength of the upper limbs: *F* = 0.29, p = 0.60, η_p_^2^ = 0.02; and cardiorespiratory fitness: *F* = 0.10, p = 0.75, η_p_^2^ = 0.01.

The post-training results showed no significant differences between groups in the body mass index (*F* = 1.40, p = 0.25, η_p_^2^ = 0.07), fat-free mass (*F* = 0.59, p = 0.45, η_p_^2^ = 0.03), hip circumference (*F* = 1.66, p = 0.21, η_p_^2^ = 0.08) and waist/hip ratio (*F* = 0.01, p = 0.92, η_p_^2^ = 0.01). Despite no significant differences were found, body mass (*F* = 2.57, p = 0.13, η_p_^2^ = 0.12), fat mass percentage (*F* = 3.61, p = 0.07, η_p_^2^ = 0.17) and waist circumference (*F* = 2.69, p = 0.12, η_p_^2^ = 0.12) showed moderated effect sizes between Exercise and Control after intervention.

Regarding lipid profile, no significant differences between groups were found in post-training for cholesterol (*F* = 1.04, p = 0.32, η_p_^2^ = 0.05), triglycerides (*F* = 3.84, p = 0.07, η_p_^2^ = 0.19), systolic blood pressure (*F* = 3.12, p = 0.09, η_p_^2^ = 0.14), and diastolic blood pressure (*F* = 0.18, p = 0.68, η_p_^2^ = 0.01). Nevertheless, triglycerides and systolic blood pressure showed moderate effect sizes between Exercise and Control.

We have presented the results of our statistical analysis between groups after the intervention, considering the adjustment for age and baseline values. However, a deeper analysis was performed to verify the effects within groups. To this end, the following results are unadjusted, analyzing each variable change from baseline following the intervention, presenting absolute and relative values, changes, 95% CI, effect sizes and p-values for within groups comparison.

**Fig 1:**
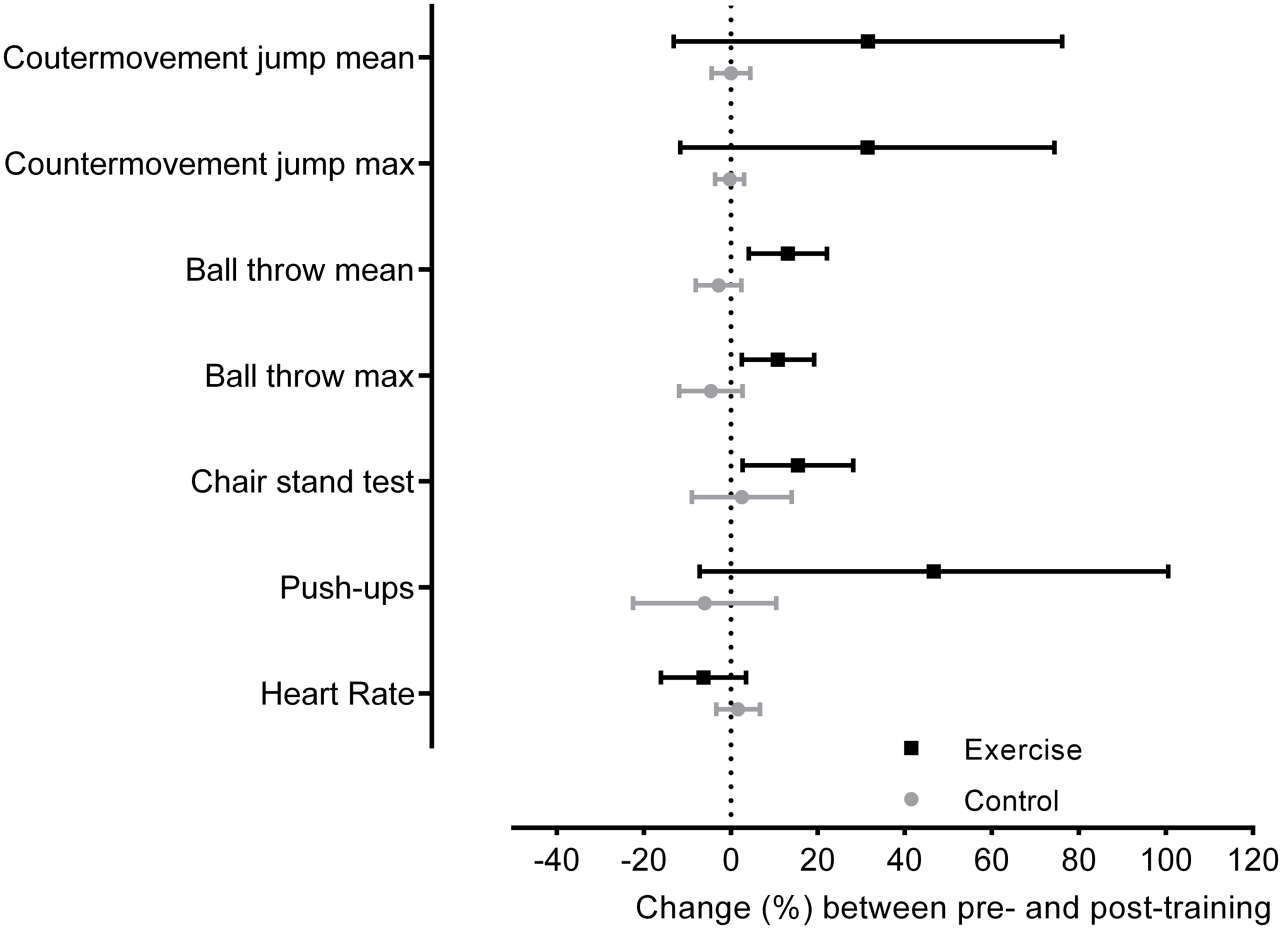
Physical condition changes between pre and post training. Mean changes and 95% confidence intervals between the initial (pre) and final (post) evaluation of physical condition variables in the Exercise and Control.

The water aerobics program enhanced the explosive strength gains in the lower and upper limbs in 10 and 11 of the 15 subjects, respectively. Most of the subjects in the Exercise showed increased endurance strength in the lower limbs (n = 11). These favorable effects on the physical condition of those who practiced a 12-week program of water aerobics can be verified in Table 1. Among these results, the significant gains in the explosive strength of the upper and lower limbs in the Exercise are worth noting (Fig 1).

**Table 1:** Physical condition values of the Control and Exercise in pre and post-training.

	Control (n = 8)				Exercise (n = 15)			
	Pre-training	Post-training	Change (95%CI)	*p* value (ES)	Pre-training	Post-training	Change (95%CI)	*p* value (ES)
CMJ mean [cm]	12.01 ± 5.49	11.93 ± 5.18	-0.09 (-0.70, 0.52)	0.74 (0.12)	7.82 ± 2.46	9.32 ± 3.26	1.50 (-0.22, 3.22)	0.08 (0.51)
CMJ max [cm]	12.91 ± 5.51	12.86 ± 5.43	-0.05 (-0.49, 0.39)	0.80 (0.09)	8.28 ± 2.53	9.89 ± 3.41	1.64 (-0.21, 3.50)	0.08 (0.50)
BT mean [m]	2.77 ± 0.78	2.70 ± 0.82	-0.07 (-0.23, 0.09)	0.33 (0.37)	2.40 ± 0.50	2.66 ± 0.48	0.26 (0.03, 0.49)	0.03 (0.63)
BT max [m]	2.89 ± 0.76	2.77 ± 0.88	-0.12 (-0.37, 0.13)	0.30 (0.40)	2.53 ± 0.52	2.77 ± 0.53	0.23 (0.01, 0.46)	0.04 (0.58)
CST [reps]	19.13 ± 4.12	19.13 ± 2.36	0.00 (-2.53, 2.53)	1.00 (0.00)	16.60 ± 4.01	18.53 ± 3.58	1.93 (-0.32, 4.19)	0.09 (0.47)
Push-ups [reps]	10.43 ± 2.51	9.71 ± 2.63	-0.71 (-2.90, 1.47)	0.45 (0.28)	6.60 ± 5.38	8.20 ± 7.07	1.60 (-2.34, 5.54)	0.38 (0.29)
HR [bpm]	98 ± 9	99 ± 7	1.25 (-4.33, 6.83)	0.69 (0.15)	106 ± 16	98 ± 16	-8.36 (-21.10, 4.38)	0.18 (0.38)

Values are presented as mean ± SD, mean and 95% confidence interval (lower CI, upper CI) for the change from pre- to post-training of countermovement jump (CMJ), mean and maximum (max), ball throw (BT), mean and maximum (max), chair stand test (CST), push-ups, and heart rates after YMCA test (HR). The p-values and effect sizes (ES) between the initial (pre-training) and final (post-training) moments are presented.

**Fig 2:**
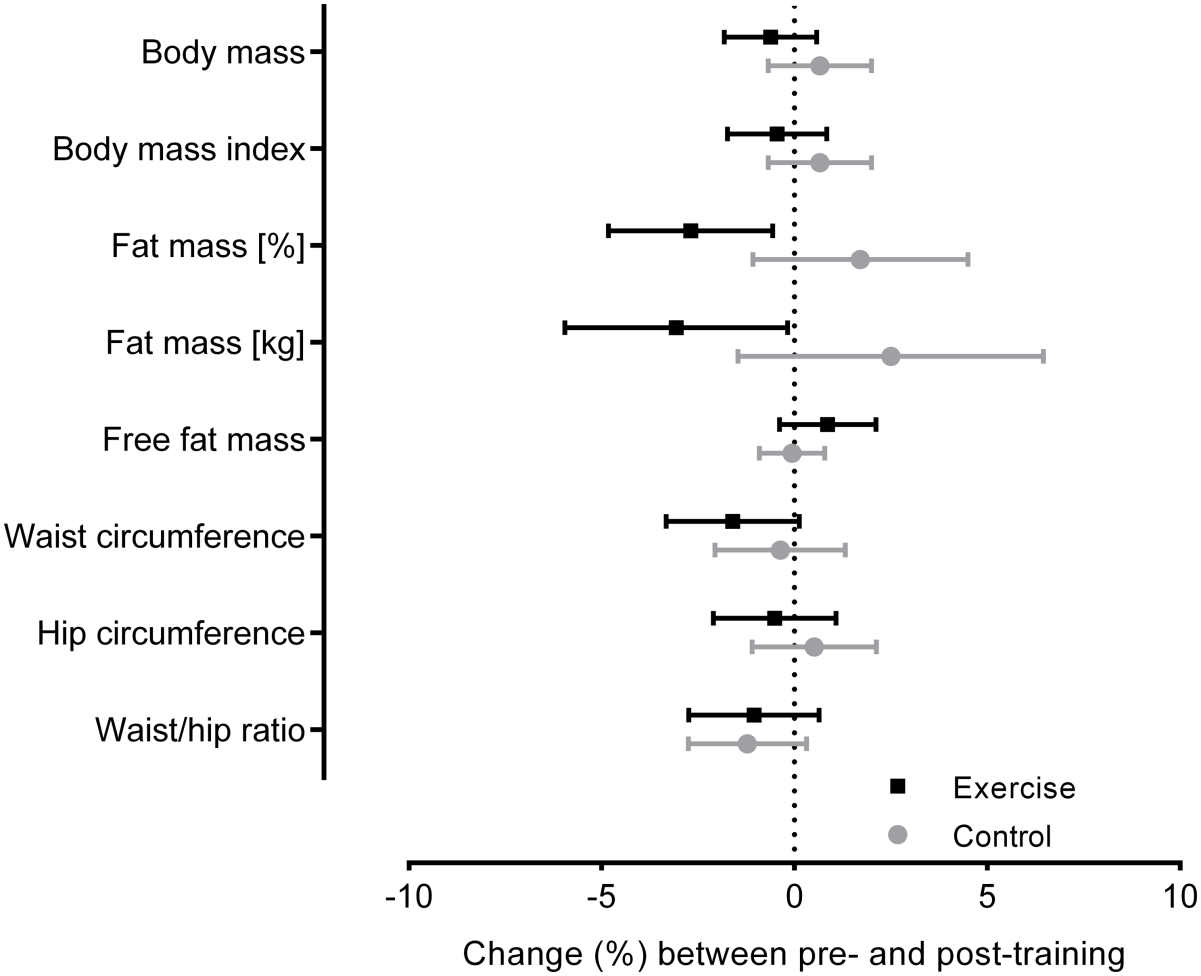
Anthropometric changes between pre and post- training. Mean changes and 95% confidence interval between the initial (pre) and final (post) evaluation of anthropometric variables in the Exercise and Control.

Table 2 shows the values for the anthropometrics variables assessed at baseline and after 12 weeks of water aerobics. When verifying the changes over the 12 weeks, no significant differences were found in the Control between the pre- and post-training moments. However, in the Exercise, there was a significant decrease in fat mass that caused a moderate decrease in body mass (Fig 2). Only five subjects in the Exercise did not lose body mass, and four of them did not decrease in fat mass.

**Table 2:** Anthropometric values of the Control and Exercise in pre and post-training testing.

	Control (n = 8)				Exercise (n = 15)			
	Pre-training	Post-training	Change (95%CI)	*p* value (ES)	Pre-training	Post-training	Change (95%CI)	*p* value (ES)
BM [kg]	70.71 ± 8.66	71.10 ± 8.09	0.39 (-0.76, 1.54)	0.45 (0.28)	72.54 ± 15.53	71.88 ± 14.29	-0.66 (-1.67, 0.35)	0.18 (0.53)
BMI [kg/m^2^]	27.07 ± 2.80	27.22 ± 2.51	0.15 (-0.31, 0.61)	0.46 (0.28)	27.75 ± 5.21	27.56 ± 4.79	-0.19 (-0.60, 0.21)	0.33 (0.23)
FM [%]	29.80 ± 5.77	30.30 ± 5.79	0.50 (-0.53, 1.53)	0.29 (0.40)	35.44 ± 7.07	34.56 ± 7.21	-0.89 (-1.74, -0.03)	0.04 (0.61)
FM [kg]	21.04 ± 5.02	21.46 ± 4.54	0.42 (-0.58, 1.41)	0.35 (0.35)	26.79 ± 10.30	25.83 ± 9.39	-0.96 (-2.08, 0.17)	0.09 (0.58)
FFM [kg]	49.67 ± 7.88	49.65 ± 7.94	-0.02 (-0.50, 0.46)	0.93 (0.03)	46.82 ± 6.76	47.17 ± 6.61	0.36 (-0.24, 0.95)	0.22 (0.37)
WC [cm]	95.50 ± 7.48	95.13 ± 7.45	-0.37 (-2.37, 1.62)	0.67 (0.16)	93.80 ± 12.12	92.07 ± 9.99	-1.73 (-3.76, 0.30)	0.09 (0.45)
HC [cm]	105.13 ± 5.25	105.63 ± 5.01	0.50 (-1.55, 2.55)	0.58 (0.20)	103.50 ± 11.79	102.80 ± 10.23	-0.70 (-2.52, 1.12)	0.42 (0.23)
W/H ratio	0.91 ± 0.05	0.90 ± 0.05	-0.01 (-0.03, 0.01)	0.17 (0.54)	0.91 ± 0.06	0.90 ± 0.05	-0.01 (-0.03, 0.01)	0.26 (0.31)

Values are presented as mean ± SD, mean and 95% confidence interval (lower CI, upper CI) for the change from pre- to post-training of body mass (BM), body mass index (BMI), fat mass (FM), free fat mass (FFM), waist circumference (WC), hip circumference (HC) and waist to hip ratio (W/C ratio). The p-values and effect sizes (ES) between the initial (pre-training) and final (post-training) moments are presented.

**Fig 3:**
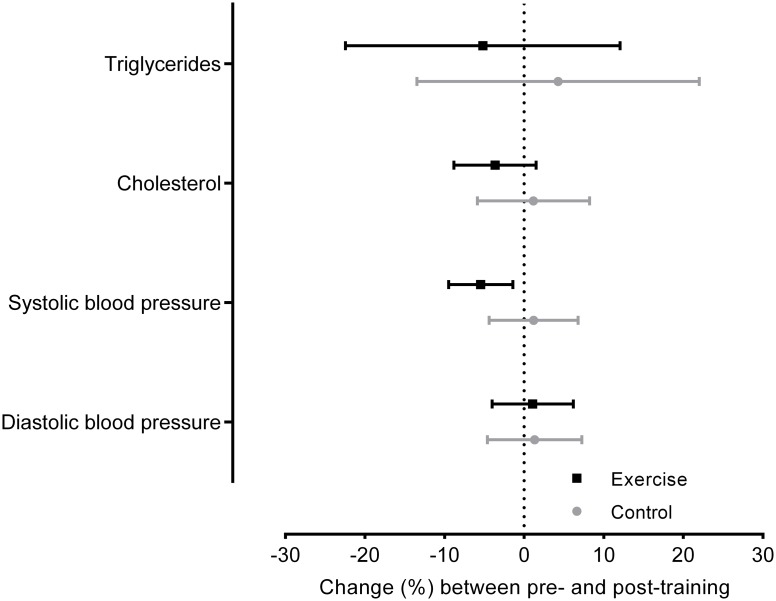
Lipid profile and blood pressure changes between pre- and post- training. Mean changes and 95% confidence intervals between the initial (pre) and final (post) evaluations of lipid profile and blood pressure in the Exercise and Control.

Table 3 shows the values obtained at the two evaluation moments for triglycerides, cholesterol and blood pressure (systolic and diastolic). It should be noted that the values appear to have moderately changed in the Exercise, with systolic blood pressure revealing a marked decrease between time points. Despite no significant effects were observed on triglycerides and cholesterol, we should emphasize that there was a trend toward reduced values in the Exercise, which is more clear in Fig 3. Of the 15 subjects, nine benefited from the water aerobics program in terms of triglycerides, and 10 benefited in terms of cholesterol values.

**Table 3:** Lipid profile and blood pressure values of the Control and Exercise in pre and post-training.

	Control (n = 8)				Exercise (n = 15)			
	Pre-training	Post-training	Change (95%CI)	*p* value (ES)	Pre-training	Post-training	Change (95%CI)	*p* value (ES)
Triglycerides [mg / dl]	175.75 ± 74.81	175.88 ± 64.35	0.12 (-31.60, 31.85)	0.99 (0.003)	160.33 ± 116.17	134.83 ± 60.53	-25.50 (-73.63, 22.63)	0.27 (0.34)
Cholesterol [mg / dl]	221.38 ± 27.69	223.25 ± 27.90	1.87 (-15.44, 19.19)	0.80 (0.09)	206.27 ± 26.97	199.20 ± 35.80	-7.07 (-18.98, 4.85)	0.22 (0.33)
Systolic BP [mmHg]	13.10 ± 1.55	13.18 ± 1.08	0.07 (-0.90, 1.05)	0.86 (0.06)	13.57 ± 1.95	12.74 ± 1.48	-0.83 (-1.46, -0.19)	0.01 (0.71)
Diastolic BP [mmHg]	7.83 ± 0.54	7.90 ± 0.51	0.07 (-0.46, 0.61)	0.75 (0.12)	7.59 ± 1.05	7.63 ± 0.97	0.04 (-0.39, 0.47)	0.84 (0.05)

Values are presented as mean ± SD, mean and 95% confidence interval (lower CI, upper CI) for the change from pre- to post-training of triglycerides, cholesterol, systolic blood pressure (Systolic BP) and diastolic blood pressure (Diastolic BP). The p-values and effect sizes (ES) between the initial (pre-training) and final (post-training) moments are presented. To convert triglycerides to mmol/L divide by 88.57. To convert cholesterol to mmol/L divide by 38.67.

## Discussion

The main purpose of this study was to verify the effects of a 12-week water aerobics program on physical fitness (explosive and strength endurance of the lower and upper limbs, cardiorespiratory fitness) and health status variables (body anthropometrics, lipid profile and blood pressure) in adults, on a real-life context of sports academy. The results showed that 12 weeks of water aerobics greatly improved the explosive strength of the upper limbs. Moderate improvements were found in the explosive and endurance strength of the lower limbs. Therefore, we accept the main research hypothesis that 12 weeks of water aerobics provide an adequate stimulus to increase explosive and endurance strength, but reject the hypothesis of cardiorespiratory improvement. In addition, the Exercise decreased body fat and decreased systolic blood pressure, without changing lipid profile values. Thus, it appears that a 12-week water aerobics program (two 50-minute classes per week) is enough to induce improvements in explosive strength, body composition, and blood pressure in the adult population.

### Physical condition

There was a tendency towards improved explosive and endurance strength in the Exercise after water training, with better results for the explosive strength of the upper limbs. Similarly, after 12 weeks of water aerobics, there was a significant (40%) increase in the explosive strength of the knee flexors and extensors for a group of older women [11]. These improvements could be explained by the overload provided by the water combined with movements performed at high velocities [25]. In our specific case, there was a greater increase in the upper limbs, perhaps because the exercises performed stimulated rapid movements of these limbs. A previous study showed that aquatic resistance training resulted in significant improvements in the muscle torque and neural activation of the trained muscles. [26]. However, some programs were not effective enough to produce a significant increase in explosive strength in older women [27]. The different intensities, in which the velocity of the movement is the main factor, might explain these differences. It is known that when an individual spends a long time performing the same exercise movement in the water, there is less recruitment of motor units, whereas the same exercise performed for a shorter duration allows higher movement velocity, causing an increase in motor unit recruitment and creating more appropriate circumstances for force production [25].

In addition to a moderate increase in the explosive strength of the lower limbs, there were slight improvements in strength endurance. Both these results could have been caused by the regular and repeated leg actions required by the exercises used in the program. This includes the usual “little jumps” that are performed even when exercises are focused on the upper limbs and trunk. For increased gains in the endurance strength of the lower limbs, participants should engage in a program with a considerably longer duration, as suggested [28], or focus on that particular type of resistance exercise [26]. Moreover, the intensity of the stimulus provided should be sufficient to cause changes; however, such guidelines are not well defined in the literature [18].

Cardiorespiratory fitness also seems to require more time to adapt to the point that the results will be clearly relevant. Recent studies have shown that long periods of water aerobics classes (24 weeks) were needed to produce an increase of approximately 10% in cardiorespiratory capacity [29]. In obese subjects, this fact is evidenced by findings that short-term water aerobics programs are not effective for improving cardiorespiratory capacity [30]. In addition to questions regarding the duration of the programs, it is difficult to find valid and reliable methods for evaluating cardiorespiratory capacity in exercise programs conducted in the water, resulting in different forms of evaluation and consequently different results. However, previous studies are consistent in their report of aerobic capacity improvements in terms of oxygen uptake, suggesting that water-based exercise performed at moderate to high intensities has positive results. In fact, intensity used should be a major issue when discussing results. Existing studies provide meager clear information about intensity, which could influence the results obtained. When data on intensity were provided, suggested ranges were between 60 and 85% of the age-predicted heart rate [18]. Therefore, our program determined that in a real-life context and venue and with older adults, the intensity of exercise was at the lower limit of this range, which may have limited the results.

### Anthropometrics

Body composition was improved after 12 weeks of water aerobics in the Exercise, mainly due to the loss of fat mass; however, there were no differences in the Control. In addition, the Exercise presented a small decrease in the hip circumference. These findings seem to be in accordance with previous findings for an aquatic activity program with the same duration [11, 14, 31] and with longer durations [32–34]. Those studies showed positive changes in body composition resulting from a 4 to 9% reduction in the participants’ fat mass, with better results for longer interventions. Moreover, a previous study of a 12-week intervention showed significant reductions in the waist/hip ratio [14], with greater improvements than those identified in the present investigation. It is possible that the longer duration of each water aerobics session in that study (10 min longer than ours) could explain the greater differences reported. In fact, duration could be barrier against improvements in anthropometrical variables. Short-term water aerobics programs (8 to 12 weeks) were unable to produce significant changes in body composition in elderly women or obese individuals, specifically in terms of weight reduction and body circumferences [30]. However, others have observed that long-term water aerobics programs (8 months) provided a considerable reduction in waist and hip circumferences in adults and elderly women [35]. This trend emphasized that to promote anthropometric changes in body circumferences, the duration of the programs should probably be longer than the 12 weeks used in the present study. Nevertheless, there is no agreement about the time needed for positive results. The contradictory results between studies might be due to differences in the intensity of the classes or in the individuals who participated in the program.

### Lipid profile and blood pressure

It was verified that a water aerobics program lasting for 12 weeks reduced systolic blood pressure but not diastolic blood pressure. These results are in agreement with previous investigations that focused on the acute effects of water exercise [36, 37]. Long-term adaptations were found after 24 weeks of a water-based intervention, with positive effects on systolic blood pressure and resting heart rates [29]. These positive results were explained by the exercise itself and because of the high temperature usually found in the swimming pools. Previous research showed that when older adults practice water aerobics in an aquatic environment with high temperatures (36°C), there is a decrease in diastolic and systolic blood pressure [38]. Perhaps there was an adaptation of the sympathetic and parasympathetic nervous system, with the reduction of the first and stimulation of the second, a change that has already been shown when the bodies are immersed into higher temperatures [39]. It would be interesting to suggest that the high temperature by itself could lead to a reduction in the blood pressure of hypertensive individuals. However, this assertion would require additional longitudinal investigations. It is worth noting the positive effect that water aerobics had on reducing systolic blood pressure. This can be considered quite relevant since it seems more difficult to Control systolic blood pressure than diastolic blood pressure through medication [40]. In addition, systolic blood pressure more accurately predicts future coronary heart disease in people over 50 years old [41]. In this sense, it seems that exercise programs performed in an aquatic environment are a great alternative for hypertensive individuals.

Regarding the lipid profile, only small decreases were found in cholesterol and triglycerides in the Exercise. It is possible that the 12-week duration and/or the intensity used were not totally efficient since the positive effects of physical exercise on the regulation of lipoprotein metabolism are well known and well documented [42]. Unfortunately, few studies have focused on the particular case of exercise in an aquatic environment. Contrary to our results, previous authors found a 4% to 6% decrease in cholesterol values [43, 44] and a 4% decrease in triglycerides [44] in longitudinal studies on water aerobics. Our results were not as clear as those of previous studies, possibly due to the lower intensity [43] and/or the shorter duration of the exercise performed [44]. The slightly positive changes observed in the lipid profile may be the effect of the beneficial impact of physical activity not only on body mass but also on body composition [14], suggesting that some reductions in the lipid profile are also due to the reduction of body fat. Thus, it seems evident that the intensity and duration of aquatic activity programs might be a key determinant of their beneficial effects on lipid profiles, as is the case for body fat reduction.

The present study took a novel approach to examining water aerobics programs in an attempt to study real cases that were ecologically valid, taught by a teacher with autonomy within the program and included the usual participants. This allowed us to investigate the practice in a real context to the greatest degree possible, verifying and analyzing the real adaptations and this is one of strength of our study. Nevertheless, some limitations that result from such context should be considered, including the program and methods used by the teacher and some nutritional feedback. Another limitation is the small and unbalanced sample sizes for each group. Further studies should include a larger number of participants to clarify some of the analyzed findings. However, we took a number of steps to strengthen our statistical analysis as described in the statistical section. Moreover, other evaluation methods could be used to complement our measures and to deepen our findings, such as breath-by-breath measurement of oxygen to assess cardiorespiratory fitness applied in a progressive protocol, and/or including skinfolds measurements for body anthropometric evaluation. Considering our limitations, readers should interpret our results with discernment. Even so, the current findings still relevant for adults and the older adults, who depend greatly on both strength and cardiovascular fitness for ongoing quality of life.

## Conclusion

The current results suggest that a 12-week water aerobics program held twice a week for 50 min per session contributes favorably to improve explosive strength, especially of the upper limbs. Moreover, it reduces the body fat mass and the systolic blood pressure. However, it does not appear to cause significant changes in the lipid profile of adults and older adults. Moreover, in a real-life context, lessons seem to be performed with low intensity loads, a finding that could provide relevant information for professionals and researchers. Sessions should be prescribed and performed with sufficient intensity to optimize the stimulus, especially for strength exercises. Further high-quality studies with ecological validity should be performed to better determine the effects of the methods implemented and to optimize the benefits of this physical activity, which is increasingly being practiced today.

## References

[1] Raffaelli C, Milanese C, Lanza M, Zamparo P. Water-based training enhances both physical capacities and body composition in healthy young adult women. Sport Sci for Health. 2016; 12(2): 1–13.

[2] Alberton L, Antunes H, Beilke D, Pinto S, Kanitz C, Tartaruga P, et al Maximal and ventilatory thresholds of oxygen uptake and rating of perceived exertion responses to water aerobic exercises. J Strength Cond Res. 2013; 27(7): 1897–1903. doi: 10.1519/JSC.0b013e3182736e47 23037612 10.1519/JSC.0b013e3182736e47

[3] Kutzner I, Richter A, Gordt K, Dymke J, Damm P, Duda GN, et al Does aquatic exercise reduce hip and knee joint loading? In vivo load measurements with instrumented implants. PLoS ONE. 2017; 12(3): e0171972 doi: 10.1371/journal.pone.0171972 28319145 10.1371/journal.pone.0171972 PMC5358747

[4] Rica L, Carneiro M, Serra J, Rodriguez D, Junior P, Francisco L, et al Effects of water-based exercise in obese older women: Impact of short-term follow-up study on anthropometric, functional fitness and quality of life parameters. Geriatr Gerontol Int. 2012; 13(1): 209–214. doi: 10.1111/j.1447-0594.2012.00889.x 22694304 10.1111/j.1447-0594.2012.00889.x

[5] Borreani S, Colado C, Calatayud J, Pablos C, Moya-Nájera D, Triplett T. Aquatic resistance training: Acute and chronic effects. Strength & Cond J. 2014; 36(3): 48–61.

[6] Barbosa TM, Marinho DA, Reis VM, Silva AJ, Bragada JA. Physiological assessment of head-out aquatic exercises in healthy subjects: a qualitative review. J Sports Sci Med. 2009; 8(2): 179–189. 24149524 PMC3761490

[7] Benelli P, Ditroilo M, Vito G. Physiological responses to fitness activities: a comparison between land-based and water aerobics exercise. J Strength Cond Res. 2004;18(4): 719–722. doi: 10.1519/14703.1 15574073 10.1519/14703.1

[8] Butts N, Tucker M, Smith R. Maximal responses to treadmill and deep-water running in high school female cross country runners. Res Q Exerc Sport. 1991; 62: 236–239. doi: 10.1080/02701367.1991.10608716 1925049 10.1080/02701367.1991.10608716

[9] Colado JC, Tella V, Triplett NT, González LM. Effects of a short-term aquatic resistance program on strength and body composition in fit young men. J Strength Cond Res. 2009; 23: 549–559. doi: 10.1519/JSC.0b013e31818eff5d 19204568 10.1519/JSC.0b013e31818eff5d

[10] Broman G, Quintana M, Lindberg T, Jansson E, Kaijser L. High intensity deep water training can improve aerobic power in elderly women. Eur J Appl Physiol. 2006; 98(2): 117–123. doi: 10.1007/s00421-006-0237-2 16924529 10.1007/s00421-006-0237-2

[11] Takeshima N, Rogers ME, Watanabe E, Brechue WF, Okada A, Yamada T, et al Water-based exercise improves health-related aspects of fitness in older women. Med Sci Sports Exerc. 2002; 34(3): 544–551. 11880822 10.1097/00005768-200203000-00024

[12] PÖyhÖnen T, SipilÄ S, Keskinen KL, Hautala A, Savolainen J, MÄlkiÄ E. Effects of aquatic resistance training on neuromuscular performance in healthy women. Med Sci Sports Exerc. 2002; 34(12): 2103–2109. doi: 10.1249/01.MSS.0000039291.46836.86 12471323 10.1249/01.MSS.0000039291.46836.86

[13] Tsourlou T, Benik A, Dipla K, Zafeiridis A, Kellis S. The effects of a twenty-four-week aquatic training program on muscular strength performance in healthy elderly women. J Strength Cond Res. 2006; 20(4): 811–818. doi: 10.1519/R-18455.1 17194242 10.1519/R-18455.1

[14] Irandoust K, Taheri M. The effects of aquatic exercise on body composition and nonspecific low back pain in elderly males. J Phys Ther Sci. 2015; 27(2): 433–435. doi: 10.1589/jpts.27.433 25729184 10.1589/jpts.27.433 PMC4339154

[15] Duvivier BM, Schaper NC, Bremers MA, van Crombrugge G, Menheere PP, Kars M et al Minimal intensity physical activity (standing and walking) of longer duration improves insulin action and plasma lipids more than shorter periods of moderate to vigorous exercise (cycling) in sedentary subjects when energy expenditure is comparable. PLoS ONE. 2013; 8(2): e55542 doi: 10.1371/journal.pone.0055542 23418444 10.1371/journal.pone.0055542 PMC3572053

[16] Volaklis KA, Spassis AT, Tokmakidis SP. Land versus water exercise in patients with coronary artery disease: effects on body composition, blood lipids, and physical fitness. Am Heart J. 2007; 154(3): 560–e1. doi: 10.1016/j.ahj.2007.06.029 17719306 10.1016/j.ahj.2007.06.029

[17] Delevatti R, Marson E, Kruel LF. Effect of aquatic exercise training on lipids profile and glycaemia: a systematic review. Revista Andaluza de Medicina del Deporte. 2015; 8(4): 163–170. doi: 10.1016/j.ramd.2014.08.003

[18] Bergamin M, Zanuso S, Alvar BA, Ermolao A, Zaccaria M. Is water-based exercise training sufficient to improve physical fitness in the elderly?. Eur Rev Aging Phys Act. 2012; 9(2): 129–141. doi: 10.1007/s11556-012-0097-1

[19] Mayhew JL, Ware JS, Johns RA, Bemben MG. Changes in upper body power following heavy-resistance strength training in college men. Int J Sports Med. 1997;18: 516–520. doi: 10.1055/s-2007-972674 9414074 10.1055/s-2007-972674

[20] Rikli E, Jones J. Functional fitness normative scores for community-residing older adults, ages 60–94. J Aging Phys Act. 1999;7: 162–181.

[21] American College of Sports Medicine. ACSM's guidelines for exercise testing and prescription. 9th ed Philadelphia: Wolters Kluwer, Lippincott Williams & Wilkins; 2013.

[22] Stewart A, Marfell-Jones M. International Standards for Anthropometric Assessment. Lower Hutt, New Zealand: International Society for the Advancement of Kinanthropometry; 2011.

[23] Gallagher D, Heymsfield SB, Heo M, Jebb SA, Murgatroyd PR, Sakamoto Y. Healthy percentage body fat ranges: an approach for developing guidelines based on body mass índex. Am J Clin Nutr. 2000;72(3): 694–701. doi: 10.1093/ajcn/72.3.694 10966886 10.1093/ajcn/72.3.694

[24] Lakens D. Calculating and reporting effect sizes to facilitate cumulative science: A practical primer for t-tests and ANOVAs. Front Psychol. 2013;4: 863 doi: 10.3389/fpsyg.2013.00863 24324449 10.3389/fpsyg.2013.00863 PMC3840331

[25] Graef FI, Pinto RS, Alberton CL, Lima WC, Kruel LF. The effects of resistance training performed in water on muscle strength in the elderly. J Strength & Cond Res. 2010;24(11): 3150–3156. 20940648 10.1519/JSC.0b013e3181e2720d

[26] Poyhonen T, Sipila S, Keskinen KL, Hautala A, Savolainen J, Malkia E. Effects of aquatic resistance training on neuromuscular performance in healthy Women. Med Sci Sports Exerc. 2002;34(12): 2103–2109. doi: 10.1249/01.MSS.0000039291.46836.86 12471323 10.1249/01.MSS.0000039291.46836.86

[27] Taunton E, Rhodes C, Wolski A, Donelly M, Warren J, Elliot J, et al Effects of land-based and water-based fitness programs on the cardiovascular fitness, strength and flexibility of women aged 65–75 years. Gerontology. 1996;42(4): 204–210. doi: 10.1159/000213794 8832268 10.1159/000213794

[28] Cardoso AS, Mazo GZ, Balbé GP. Strength levels of aged women practicing water-based exercises: A two-year follow-up. Motriz. 2016;16(1): 86–94. doi: 10.5016/1980-6574.2010v16n1p86

[29] Piotrowska-Calka E. Effects of a 24-week deep water aerobic training program on cardiovascular fitness. Biol Sport. 2010;27(2): 95–98.

[30] Penaforte FRO, Calhau R, Mota GR, Chiarello PG. Impact of short-term water exercise programs on weight, body composition, metabolic profile and quality of life of obese women. J Hum Sport Exerc. 2015; 10 (4): 915–926.

[31] Kasprzak Z, Pilaczynska-Szczesniak Ł. Effects of regular physical exercises in the water on the metabolic profile of women with abdominal obesity. J Hum Kinet. 2014;41(1): 71–79. 25114733 10.2478/hukin-2014-0034 PMC4120466

[32] Bergamin M, Ermolao A, Tolomio S, Berton L, Sergi G, Zaccaria M. Water-versus land-based exercise in elderly subjects: effects on physical performance and body composition. Clin Interv Aging. 2013; 8:1109–17. doi: 10.2147/CIA.S44198 24009416 10.2147/CIA.S44198 PMC3762608

[33] Gappmaier E, Lake W, Nelson AG, Fisher AG. Aerobic exercise in water versus walking on land: effects on indices of fat reduction and weight loss of obese women. J Sports Med Phys Fitness. 2006; 46(4): 564–569. 17119521

[34] Vedana TA, Santos RN, Pereira JM, Araujo SP, Portes Júnior MP, Portes LA. Shallow-water exercise influence upon body composition, cardiovascular, hematological, spirometry and fitness of adults and older women and men. Brazilian Journal of Biomotricity. 2011;5 (2): 65–79.

[35] Gubiani GL, Neto CSP, Petroski ÉL, da Silva Lopes A. Effects of water gymnastics on anthropometric variables of women aged 60–80 years. Braz. J Kinathrop Hum Perform. 2001;3(1): 34–41.

[36] Cunha M, Arsa G, Neves B, Lopes C, Santana F, Noleto V, et al Water aerobics is followed by short-time and immediate systolic blood pressure reduction in overweight and obese hypertensive women. J Am Soc Hypertens. 2016;10(7): 570–577. doi: 10.1016/j.jash.2016.05.002 27245928 10.1016/j.jash.2016.05.002

[37] Terblanche E, Millen AM. The magnitude and duration of post-exercise hypotension after land and water exercises. Eur J Appl Physiol. 2012;112(12): 4111–4118. doi: 10.1007/s00421-012-2398-5 22526248 10.1007/s00421-012-2398-5

[38] Bergamin M, Ermolao A, Matten S, Sieverdes C, Zaccaria M. Metabolic and cardiovascular responses during aquatic exercise in water at different temperatures in older adults. Res Q Exerc Sport. 2015;86(2): 163–171. doi: 10.1080/02701367.2014.981629 25513937 10.1080/02701367.2014.981629

[39] Nahimura K, Yianishi A, Komiyama M, Yoshioka A, Seki K, Ono, et al. Effects of immersion in different water temperature before exercise on heart rate, cardiac parasympathetic nervous system and rectal temperature. In: Nomura T, Ungerechts B.E, editors. The Book of Proceedings of the 1st International Scientific Conference of Aquatic Space Activities. Tskuba: University of Tskuba; 2008. pp. 128–133.

[40] Lloyd-Jones DM, Evans JC, Larson MG, O’donnell CJ, Roccella EJ, Levy D. Differential control of systolic and diastolic blood pressure. Hypertension. 2000;36(4): 594–599. 11040241 10.1161/01.hyp.36.4.594

[41] Zanchetti A, Waeber B. Hypertension: which aspects of hypertension should we impact on and how? J Hypertens. 2006;24 (Suppl 5): S2–S5. doi: 10.1097/01.hjh.0000240039.97472.f1 16936532 10.1097/01.hjh.0000240039.97472.f1

[42] Kelley GA, Kelley KS, Tran ZV. Walking, lipids, and lipoproteins: a meta-analysis of randomized controlled trials. Prev Med. 2004;38(5): 651–661. doi: 10.1016/j.ypmed.2003.12.012 15066369 10.1016/j.ypmed.2003.12.012

[43] Kantyka J, Herman D, Roczniok R, Kuba L. Effects of aqua aerobics on body composition, body mass, lipid profile, and blood count in middle-aged sedentary women. Hum Mov. 2015;16(1): 9–14. doi: 10.1515/humo-2015-0020

[44] Van Roie E, Delecluse C, Opdenacker J, De Bock K, Kennis E, Boen F. Effectiveness of a lifestyle physical activity versus a structured exercise intervention in older adults. J Aging Phys Act. 2010;18(3): 335–352. 20651418 10.1123/japa.18.3.335

